# Transformer-Based Deep Neural Language Modeling for Construct-Specific Automatic Item Generation

**DOI:** 10.1007/s11336-021-09823-9

**Published:** 2021-12-14

**Authors:** Björn E. Hommel, Franz-Josef M. Wollang, Veronika Kotova, Hannes Zacher, Stefan C. Schmukle

**Affiliations:** 1 Department of Work and Organizational Psychology, Institute of Psychology – Wilhelm Wundt, Leipzig University, Neumarkt 9-19, Leipzig, 04109 Germany; 2 magnolia psychometrics GmbH, Leipzig, Germany; 3 Department of Informatics, Technical University of Munich, Munich, Germany

**Keywords:** automatic item generation, natural language processing, deep learning, neural networks, language modeling

## Abstract

Algorithmic automatic item generation can be used to obtain large quantities of cognitive items in the domains of knowledge and aptitude testing. However, conventional item models used by template-based automatic item generation techniques are not ideal for the creation of items for non-cognitive constructs. Progress in this area has been made recently by employing long short-term memory recurrent neural networks to produce word sequences that syntactically resemble items typically found in personality questionnaires. To date, such items have been produced unconditionally, without the possibility of selectively targeting personality domains. In this article, we offer a brief synopsis on past developments in natural language processing and explain why the automatic generation of construct-specific items has become attainable only due to recent technological progress. We propose that pre-trained causal transformer models can be fine-tuned to achieve this task using implicit parameterization in conjunction with conditional generation. We demonstrate this method in a tutorial-like fashion and finally compare aspects of validity in human- and machine-authored items using empirical data. Our study finds that approximately two-thirds of the automatically generated items show good psychometric properties (factor loadings above .40) and that one-third even have properties equivalent to established and highly curated human-authored items. Our work thus demonstrates the practical use of deep neural networks for non-cognitive automatic item generation.

**Supplementary Information:**

The online version contains supplementary material available at 10.1007/s11336-021-09823-9.

## Introduction

Research on automatic item generation (AIG) represents a promising endeavor as it allows obtaining vast numbers of items by utilizing computer technology. Although progress in this field has yielded numerous notable contributions such as generative algorithms for creating Raven’s Progressive Matrices (Wang & Su, 2015), software for the generation of multiple-choice items (Gierl et al., 2008), and the theoretical foundations of AIG (Drasgow et al., 2006), there is a dearth of methods that can be utilized for the generation of item formats typically used to assess non-cognitive constructs such as personality traits. We believe that this gap in the literature can be attributed to the special linguistic challenges posed by items used to measure non-cognitive constructs. Recently, advances in the field of deep learning and natural language processing (NLP) have made it possible to address these challenges. In his pioneering work, von Davier (2018) successfully demonstrated that personality items can be generated by training a type of recurrent neural network known as long short-term memory (LSTM) network on a set of established personality statements. Although von Davier’s model produces syntactically correct statements that resemble those typically found in questionnaires, its utility is limited as it does not permit the generation of items that are specific to a given construct. Test development, however, is always goal-oriented and intends to measure explicit knowledge, skills, abilities, or other characteristics. As stated by Gorin and Embretson (2013), “Principled item design, whether automated or not, should begin with a clear definition of the measurement target” (p. 137). Since the publication of von Davier’s article, fast-paced developments in computer science have continued to push the boundaries of what can be achieved by language modeling.

In this article, we focus on the issue of construct-specificity for non-cognitive item generation, that is, the creation of items for a predefined measurement target. We first outline and formalize the linguistic problem that requires a solution, so that construct-specific AIG can be achieved. We then offer a brief synopsis of previous language modeling techniques to illustrate the challenging problem of synthesizing semantically and syntactically valid statements that can be used to measure psychological states and traits. We highlight a relatively new group of neural networks known as Transformers (Vaswani et al., 2017) and explain why these models are suitable for construct-specific AIG and subsequently propose a method for fine-tuning such models to this task. Finally, we provide evidence for the validity of this method by comparing human- and machine-authored items with regard to their psychometric properties.

### Challenges with the Automatic Generation of Personality Items

Modern approaches to AIG for cognitive items typically rely on a three-step process (Gierl & Lai, 2015). A target knowledge, skill, or ability is first organized into a conceptual model that structures the cognitive and content-specific information required by test takers to solve problems in the desired domain. This cognitive model is subsequently used to define a formative item model, incorporating components such as item stem, response options, and placeholder elements. Items are finally assembled by combining all possible variations of options and element inputs. While these template-based AIG techniques have indisputable advantages in comparison to manual item authoring, the generation of non-cognitive item inventories (e.g., personality questionnaires) demands somewhat different approaches (Bejar, 2013).

Rating scales are frequently used for measuring non-cognitive constructs in the social and behavioral sciences, and they can be used to illustrate the difficulty of employing template-based AIG. Consider the statement “*I am the life of the party*” used in the International Personality Item Pool (IPIP; Goldberg et al., 2006) to assess individual differences in extraversion, one of the Big Five personality traits (Digman, 1990). At least two problems immediately become apparent if we would attempt to craft an item-template based on this statement. First, when examined independently, not a single word in this sentence is explicitly descriptive of extraverted behavior. Second, if “party” were regarded as an interchangeable word, the universe of meaningful alternative nouns that could replace it is quite limited. Replacing it with synonyms or closely related words would most likely render the item trivial and restrict the scale’s ability to capture variance. This example illustrates that other non-template based generation techniques may be more adequate in the case of personality items.

Before examining possible alternatives to template-based AIG techniques, we first describe requirements that must be met by such a method. We propose four criteria that a sequence of words generated by a language model must satisfy to qualify as a rating scale component. First, the latent variable of interest must be linguistically encoded in the word sequence; this is synonymous with the concept of content validity (Cronbach & Meehl, 1955). Second, the sequence must be syntactically arranged such that it reassembles the grammar of a target natural language. Third, the sequence must have certain characteristics that elicit reliable and valid responses from test takers (see Angleitner et al., 1986 for a systematic taxonomy of typical item–construct relations). Finally, generated sequences must be segmented into meaningful units of adequate length; preferably, the text of a rating scale item should be limited to a single short sentence.

Although psychometric item and scale properties are dependent on a variety of additional formal aspects, such as avoiding double negations and ambiguity (see Krosnick & Presser, 2010, for a comprehensive overview), the mentioned characteristics represent a minimum standard for personality items created with AIG techniques. The difficulty of meeting this standard consistently with AIG becomes obvious when revisiting the previously mentioned IPIP item (“*I am the life of the party*”)—a statement that requires a considerable inferential leap to identify its relationship to trait-level extraversion. Three approaches to non-template-based AIG are typically distinguished. While *syntax*- and *semantics*-based techniques employ linguistic rule-based systems (e.g., syntax trees, grammatical tagging) to generate items, *sequence-based* procedures attempt to predict new content by using linguistic units in existing data (Xinxin, 2019). Hereafter, we examine language modeling as a sequence-based non-template approach to the automatic generation of personality items.

### Language Modeling Approaches to Construct-Specific Automatic Item Generation

In principle, the problem of AIG of personality items can be posed as a language modeling problem. A language model is a function, or an algorithm for learning such a function, that captures the salient statistical characteristics of the distribution of sequences of words in a natural language, typically allowing one to make probabilistic predictions of the next word given preceding ones (Bengio, 2008). Such models are frequently employed to solve a variety of NLP tasks, such as machine translation, speech recognition, dialogue systems, and text summarization.

Throughout this paper, we consider the problem of construct-specific AIG to be the inverse problem of text summarization (Rush et al., 2015). Instead of capturing the semantic essence of a text and producing a shorter, more concise version of it, we wish to do the inverse and expand a concept expressed by a short sequence of words or even a single word (e.g., “extraversion”) into a longer text sequence that is strongly representative. This task may be regarded as concept elaboration, which in language modeling terms can be described as the conditional probability of finding the item stem $({\upiota })$—defined as a sequence of words $\left( w_{1},w_{2},\ldots ,w_{n} \right)$—for the linguistic manifestation of a given construct $({\uppsi })$ as

1 $$\begin{aligned} P\left( {\upiota } \right) =P\left( {w_{1},w_{2},\ldots ,w_{n}}~\vert ~ {\uppsi }\right) \end{aligned}$$

However, in practice generic generative language models base their word predictions not on a global latent factor corresponding to a specific abstract concept but on previously generated words, either directly or in the form of hidden state encoding contextual information (e.g., Bengio, 2008; Zellers et al., 2019). Consequently, the conditional probability of any given word $(w_{k})$ is given by the following recurrence relation, relating it to the conditional probabilities of all previous words:

2 $$\begin{aligned} P\left( w_{\left[ 1,n \right] } \right)= & {} P\left( w_{1} \right) P\left( w_{2}~\vert ~ w_{1}\right) P\left( w_{3}~\vert ~ w_{\left[ 1,2 \right] }\right) \ldots P\left( w_{n}~\vert ~ w_{\left[ 1,n-1 \right] }\right) \nonumber \\= & {} \mathop \prod \limits _{k=1}^n {P\left( w_{k}~\vert ~ w_{\left[ 1,k-1 \right] }\right) } \end{aligned}$$

To achieve concept elaboration for construct-specific AIG, one must seek to find solutions that allow Eq. 2 to approach Eq. 1 asymptotically. For the remainder of this section, we recapitulate historical developments in NLP that have led to ever more sophisticated approaches to language modeling and that eventually allowed for construct-specific AIG as presented in this paper.

#### Markov Chains and n-gram Models

When estimating conditional word probabilities, merely counting the co-occurrence of words in a given corpus does not suffice. Alone, it fails to calculate probabilities for word sequences that have not occurred previously in the corpus. Early solutions to this problem involved the use of *n-gram* models relying on the Markovian assumption that the probability of a word can be approximated by calculating the conditional probability of the *n* words preceding it (Jurafsky & Martin, 2020). While n-gram models remain in frequent use for various NLP tasks due to their simplicity, they introduce a dilemma that becomes increasingly critical for more complex chunks of text: smaller context windows (e.g., *bigram* models) result in less accurate predictions while larger n-models decrease the probability of finding any particular sequence of words in a given text, yielding missing data. Another disadvantage of *n*-gram models is their tendency to neglect any information that is not contained in the immediate neighborhood of a target word, largely disregarding some types of syntactic structures and failing to maintain semantic continuity over larger sequences. Overall, n-grams are insufficient for the purpose of concept elaboration because the task demands the consideration of broader contextual information and AIG in the domain of personality items particular requires the creation of novel statements.

#### Distributed Semantics and Word Embeddings

The notion that semantic meaning is derived from context is the central assumption of the distributional hypothesis (Harris, 1954); as famously summarized by John R. Firth: “You shall know a word by the company it keeps” (Firth, 1962, p. 11). A notable shift toward distributional semantics in the practice of language modelling took place with the advance of word embeddings as produced by models such as word2vec (Mikolov, Chen, et al., 2013a; Mikolov, Sutskever, et al., 2013b). Word embeddings represent the meaning of words by mapping them into a high-dimensional semantic space, which is achieved by evaluating neighboring context words. Originally, this was accomplished by training a binary classifier to either predict a target word based on its context words (Continuous Bag-of-Words Model) or vice versa (Continuous Skip-gram Model). For each iteration, logistic regression weights are updated to maximize the prediction. These eventually yield an n-dimensional embedding matrix in which each word in a vocabulary is represented as an embedding vector. The embedding thereby contains semantic information and one can perform mathematical operations on the word vectors to identify relationships.

For example, if the task is to find words related to “extraversion,” a model trained on an appropriate corpus can be prompted to return the k number of words showing the highest similarity to it. The similarity may be evaluated by the value of the cosine between embedding vector pairs. “Party” might show a higher relatedness to “extraversion” than to “agreeableness,” representing the higher likelihood of “party” co-occurring with “extraversion” in a corpus or other words that co-occur with “extraversion” and thus transitively increase the similarity. A major benefit of these models is the fact that they can achieve distributed semantic representations through semi-supervised learning, meaning that they require no labeled input data and rely solely on raw text. However, since each word is represented by a single point in a semantic space, word embeddings perform poorly on words that entail multiple meanings or in the case of word sequences (Camacho-Collados & Pilehvar, 2018). Similar to n-gram models, basic word embeddings do not incorporate enough contextual information to pose a viable option for the automatic generation of personality items. Embeddings have nevertheless remained central in NLP and is an integral part of many modern architectures (e.g., the transformer model, as explained in Sect. 1).

#### Recurrent Neural Networks and Long Short-Term Memory Networks

To remedy the problem of limited contextual encoding, word embeddings have successfully been used in conjunction with a variety of deep neural networks. Deep neural networks are layered architectures that extract high-level features from input data by passing information through multiple computational stages. These stages or layers consist of multiple smaller, interconnected computational units called neurons, which behave in a manner loosely analogous to their human counterparts by altering their state through a non-linear activation (Rosenblatt, 1958; Lapedes & Farber, 1988). The outputs of the neurons of each layer are variously connected to the inputs of the subsequent layer. Similar to linear regression analysis, the initial output of a single neuron is a linear function of its inputs, a *weight*, and an associated intercept referred to as the *bias* term; however, the initial output is then always fed through a so-called activation function to get the final output—often a sigmoid, making it in some ways also similar to logistic regression. The activation signal output from one neuron represents a statistical identification or recognition of an intermediate pattern in the space formed using the previous layer’s outputs as a basis. The outputs of all neurons in a layer then together become the basis of the space in which the patterns identified by the activations of each neuron in the subsequent layer reside (Montavon et al., 2011). The accuracy of the network in achieving its task is evaluated by a predefined loss function; an iterative procedure is then followed that identifies the neurons in the network responsible for the largest losses and shifts their weights some small step in the direction of the negative gradient of the loss. This stochastic gradient-descent algorithm is known as backpropagation. Finally, various classical information-theoretical measures are used to determine when to terminate the training of the model. The use of many layers helps the model create increasingly abstract and, usually, meaningful representations of the original data that then improve its overall robustness and accuracy. Since a more thorough review of deep neural networks is beyond the scope of this article, the interested reader is referred to Lapedes and Farber (1988), Nielsen (2015), and Goodfellow et al. (2016) for introductory material.

Among deep neural network architectures, recurrent neural networks (RNNs, Elman, 1990) have been particularly convenient for language modeling. Recurrent neural networks are inherently designed to perform well on sequential data, since information about previous inputs is preserved by feeding the output of the network back into itself along with new inputs. This mnemonic quality is of crucial importance for sentence generation tasks, as the probability of a given word occurring is linked to the sequence of words preceding it. Models with this property are termed autoregressive. In practice, however, simple recurrent neural networks struggle to maintain this state persistence or coherence throughout longer input sequences and tend to “forget” previous words. This phenomenon, commonly referred to as the vanishing gradient problem (Hochreiter, 1991), is discussed in detail in Bengio et al. (1994).

*Long short-term memory models* (LSTM; Hochreiter & Schmidhuber, 1997; Jozefowicz et al., 2015) expand on the recurrent neural network architecture and solve the problem of *long-distance dependencies*, namely learning the relationships between words even if they are not in close proximity. LSTMs work by passing state vectors (the output of the network from the previous step) through a specialized structure that helps the model learn what information to remember or to forget. This structure uses *gates* to determine what information to add or to remove from the state. By actively forgetting information when it becomes irrelevant and, likewise, selecting and carrying important parts of the input data through to the next step, LSTMs have shown exceptional performance in a wide variety of NLP tasks. We refer to Olah (2015) for a thorough introduction to LSTMs.

With these developments in language modeling in mind, it is reasonable that von Davier (2018) chose LSTM models for AIG and it is apparent why there could not have been fruitful attempts prior to these advances. Since von Davier’s seminal contribution, however, research in NLP has progressed substantially. Although LSTMs show better performance than traditional recurrent neural networks in long-distance dependencies, they too suffer from vanishing gradients when given particularly long sequences and tend to require large amounts of hardware resources, preventing most researchers from being able to afford training larger models.

#### Transformer Models and the Attention Mechanism

One of the most recent and arguably substantial paradigm shifts since the initial advance of distributional semantics was sparked by the introduction of the transformer model by Vaswani et al. (2017). Its model architecture holds numerous advantages when applied to sequential data such as natural language. First, sequential data can be processed in parallel by transformer models, reducing the resources required to train such a model. Sequential information (i.e., the order of words) is preserved by a process termed *positional encoding*, which engrains each word in a sentence with its intended sequential position. As a consequence, larger and more competent language models can be trained. Second, and of central importance to the design, transformer models learn through a mechanism referred to as *self-attention*. In essence, self-attention refers to the concept of determining the relevance of a word in relation to the relevance of other words in the input sequence. We provide more details on how attention is computed in the next section of this article. In particular, these two features allow the transformer model to learn long-range dependencies better than LSTMs.

Since the publication of Vaswani et al.’s (2017) paper, a plethora of transformer implementations have been released with various modifications. One typically distinguishes between *bidirectional* and *unidirectional* transformer models. Bidirectional models attempt to predict each token in a sequence by using tokens that both precede and succeed the current target. Tokens are sequences of characters in a particular vocabulary that are grouped together as a useful semantic unit (e.g. words, syllables, prefixes, punctuations, etc.; Manning et al., 2008). This makes such models suitable for tasks like binary text classification or machine translation (Camacho-Collados & Pilehvar, 2018; González-Carvajal & Garrido-Merchán, 2021). Unidirectional models, however, based their predictions of tokens in a sequence only on the set of preceding words, making them autoregressive. They are therefore sometimes referred to as *causal transformer* models and have proven themselves to be exceptionally useful in various applications in the domain of text generation.

As noted by Vaswani et al. (2017), self-attention shows better computational performance than recurrent techniques (i.e., LSTMs) when the input sequence is smaller than the dimensionality of the word representation. It has become common practice for research teams to release transformer model implementations that have been pretrained on exceedingly large general language datasets. If such a model is obtained, one can easily perform additional training on a more task-specific dataset in a process known as *fine-tuning* (Howard & Ruder, 2018). During fine-tuning, the weights of the pretrained model will shift and bias the latent features toward a better representation of the task-specific corpus. Notable releases of bi- and unidirectional transformer models include the *Bidirectional Encoder Representations from Transformers* (BERT; Devlin et al., 2018) and the Generative Pretrained Transformer (GPT; Radford et al., 2018). In early 2019, OpenAI released the GPT-2 model (Radford et al., 2019) as the largest pretrained causal language model to that date.

GPT-2 received much attention due to its unparalleled ability to perform well across several different NLP tasks, such as reading comprehension, translation, text summarization, and question answering. Furthermore, numerous examples have demonstrated GPT-2’s ability to generate long paragraphs of text that have a startling level of syntactic and semantic coherence. It is important to note that the effectiveness of GPT-2 is not due to any major modifications to the original transformer architecture, but can largely be attributed to increased processing power and the data-set used to train the model. Specifically, the model was trained on a 40-gigabyte corpus obtained by systematically scraping 8 million web documents. In total, OpenAI has released four versions of GPT-2, with the largest model possessing a 48-layer decoder block consisting of 1.5 billion parameters, embedding words in a 1600-dimensional ambient space (Radford et al., 2019).

## Proposed Method

Although pre-trained transformer models are capable of generating fairly coherent bodies of text, it is oftentimes desirable to specialize their linguistic capabilities for specific application domains. The process of applying previously attained knowledge to solve a related family of tasks is referred to as transfer learning, and is especially powerful for applications with scarce training data (Zhuang et al., 2020). The underlying assumption is that neural networks learn relatively universal representations in the early layers that are good low-level features for a large family of related tasks. The general nature of these low-level features suggests that it should be possible to reuse them for related tasks, reducing the amount of training time or data required to derive specialized models from a general one. Utilizing pre-trained transformer models for construct-specific AIG therefore requires fine-tuning them for the task of concept elaboration.

Transformer models learn by taking the positionally encoded embeddings $x_{i}$ (as explained in Sect. 0.2.2) for each token *i* of a sequence of length *n*. The length of the embedding vectors $x_{i}$, the model dimensionality, is dependent on the language model used with typical values ranging from $d = 768$ to 1,600 in the case of GPT-2. These vectors are then multiplied with weights matrices to calculate the attention vectors $z_{i}$ for each token *i*. Each element in $z_{i}$ is an attention weight that reflects the relevance of each other token in the sequence in relation to the current token *i*.

Specifically, the attention vector $z_{i}=z_{i,1},\ldots ,z_{i,n}$ for token *i* is calculated on the basis of the vectors $q_{i}=q_{i,1},\ldots ,q_{i,n}$, $k_{i}=k_{i,1},\ldots ,k_{i,n}$ and $v_{i}=v_{i,1},\ldots ,v_{i,n}$. These vectors are obtained by $x_{i}\cdot W_{q\left| k \right| v}$ where *W* are weight matrices that are randomly initialized or learned and propagated by previous layers. While $q_{i}$ can be understood as an abstraction of the input values, $k_{i}$ are respective abstractions of all other embeddings in the context with $v_{i}$ as associated values. These vectors are obtained for each token in a given sequence and the attention matrix *Z* is then based on the aggregate matrices *Q*, *K*, *V*:

3 $$\begin{aligned} Z=\upsigma \left( \frac{QK^{T}}{\sqrt{n} } \right) \cdot V \end{aligned}$$

where $\upsigma$ is a softmax transformation for each vector of the input matrix, with length of *n*. While typically $\uptau =\, 1$ is for regular softmax, it is sometimes used as a parameter to transform the probability distribution for multinomial sampling:

4 $$\begin{aligned} \upsigma \left( a \right) =\frac{e^{\frac{a}{\uptau }}}{\sum \nolimits _{i=1}^n e^{\frac{a_{i}}{\uptau }} } \end{aligned}$$

The resulting attention matrix *Z* is a square $n \times n$ matrix containing attention weights between all the input tokens in the sequence.

**Fig. 1:**
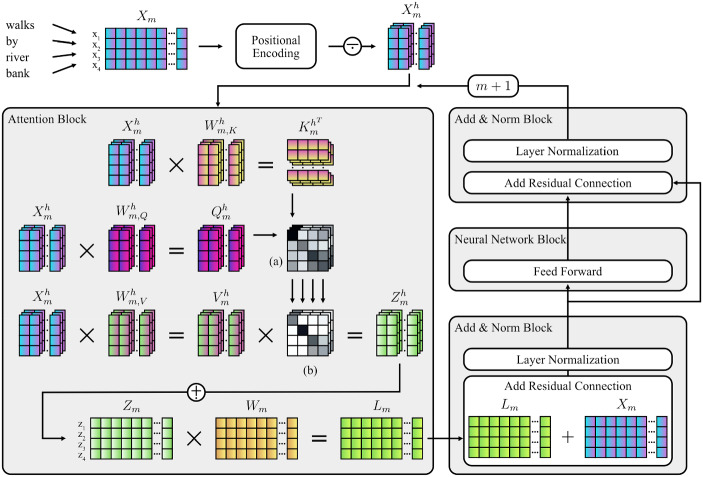
Schematic Diagram of the Attention-Mechanism and Components of the Transformer Architecture. *Note*. The process illustrates the encoding and transformation of the sequence “*walks by river bank*” by components of the transformer architecture (Vaswani et al., 2017). Weight matrices ($$W_{m,\, K\vert Q\vert V}^{h}$$ and $$W_{m})$$ are randomly initialized and then learned during the training process. In case of causal language models, masking (see Eq. 5) is applied to $$Z_{m}^{h}$$. (a) $$=$$ Matrix product of $$K_{m}^{h^{T}}$$ and $$Q_{m}^{h}$$; (b) Scaling and softmax is applied; $$n =$$ Input sequence length; $$d =$$ Model dimensionality, i.e., length of embedding vectors; *h* $$=$$ Current attention head; $$n_{h}=$$ Number of attention heads; $$m =$$ Current layer; $$X_{m}=$$ Embedding matrix ($$dimensionality:\, n\, \times d)$$; $$X_{m}^{h}=$$ Embedding matrix subset ($$n\, \times \frac{d}{n_{h}})$$; $$W_{m,\, K\vert Q\vert V}^{h} =$$ Key, query, and value weight matrices ($$n\, \times \frac{d}{n_{h}})$$; $$K_{m}^{h^{T}} =$$ Transposed key matrix ($$n\, \times \frac{d}{n_{h}})$$; $$Q_{m}^{h} =$$ Query matrix ($$n\, \times \frac{d}{n_{h}})$$; $$V_{m}^{h} =$$ Value matrix ($$n\, \times \frac{d}{n_{h}})$$; $$Z_{m}=$$ Attention matrix ($$n\, \times d)$$; $$W_{m}=$$ Weight matrix ($$n\, \times d)$$; $$L_{m}=$$ Layer output matrix ($$n\, \times d)$$;  = Matrix subdivision;  = Matrix concatenation.

In most architectures, including GPT-2, the vectors $q_{i}$, $k_{i}$, and $v_{i}$ are subdivided into multiple heads (*h*) before calculation of *Z* to allow the entire attention process described above to attend to multiple parts of the sequence at the same time; the calculation of such attention heads is repeated multiple times in parallel by concatenating the heads together into a single larger matrix. When using multiple attention heads, it becomes necessary to multiply the concatenated multi-head attention matrix by an additional final weight matrix in order to let the model learn through the training process how to map the multiple attention heads into a single homogenous attention representation. In the final step, this multi-headed self-attention matrix is subsequently normed and passed as a hidden state through a fully-connected neural network (Radford et al., 2019), before being output to the subsequent transformer layer. In this fashion, the above process repeats iteratively as embeddings are passed on through the *M* layers of the transformer (i.e., 12 to 48 layers in the case of GPT-2). Figure 1 shows a schematic depiction of the central aspects of the transformer architecture. Note that the model architecture depends on additional components, (e.g., positional encoding), which are, however, not central to this paper.

As described above, however, the attention for each token could include all other tokens in the sequence, resulting in bidirectional predictions. As previously explained, causal language models aim to predict tokens by only evaluating preceding tokens. Therefore, the self-attention must be masked to form a lower triangular matrix:

5 $$\begin{aligned} \forall z_{i,j}\in Z:j\le i\Rightarrow z_{i,j}=-\infty \end{aligned}$$

Where *i* is the position of a token in the sequence, *j* is the iteration for $j\le i$, and $-\infty$ is used rather than zeroing so that after the softmax operation the corresponding entries in the output attention vector will be zeroed.

Once training is completed, tokens can be predicted by multiplying the output vectors of the final transformer layer with the matrix of all embedding vectors *x* for the entire vocabulary and then a final softmax operation is performed to ensure that the output is a probability distribution. A sequence of words can then easily be generated either by deterministic querying or sampling by using various hyperparameters. One typically distinguishes between two generative modalities when using transformers for causal language modeling. In *unconditional* sampling, the model generates a sequence of tokens based merely on a decoding method that governs how tokens are drawn from a probability distribution. In *conditional* sampling, the output is additionally based on a fixed, predefined token or token sequence. Loosely speaking, conditional generation works by triggering the transformer models’ associations to a given input. While decoding methods permit a coarse way of controlling from what part of the probability distribution tokens are sampled, they do not grant explicit semantic output manipulation. We therefore subsequently propose a technique for the indirect parameterization of causal language models that allows for construct-specific AIG.

To leverage the capacity of pretrained language models such as GPT-2, it is conventional to perform additional training on data that is close to the target domain. In the case of AIG for personality items, the training data must naturally consist of items from validated personality test batteries. One possibility is fine-tuning models to only be capable of generating a narrow selection of items that represent a single fixed construct. Since this is an undesirable prospect, the goal must be to fine-tune a model to more generally traverse the manifold of possible item-like sequences while being guided toward specific construct-clusters. Conversely, if tokens in the beginning of a sequence are representative of a latent construct, they may be used to prompt the completion of a sentence which may also be indicative of the construct. Transformer models may then be trained to pay privileged attention to such indicative tokens. Sampling from a transformer model trained in this way would yield a closer approximation of Eq. 1. It is common practice to achieve this goal indirectly by combining special input formatting during fine-tuning with conditional text generation (e.g., Rosset et al., 2020). The special input formatting teaches the model to conform to a segmented pattern concatenated by delimiter tokens. This pattern is then partially prompted in conditional generation and extrapolated by the model output. In the context of construct-specific AIG, we propose a training pattern where ${\upphi }$ is the function encoding the construct ${\uppsi }$ and the item stem ${\upiota }$ by a concatenation ($\circ$) of strings:

6 $$\begin{aligned} {\upphi }\left( {\uppsi },{\upiota } \right) =u_{1}^{A}\circ c_{1}\circ \cdots \circ u_{m}^{A}\circ c{}_{m} \circ u^{B}\circ w_{1}\cdots w_{n} \end{aligned}$$

In this pattern, the single character delimiter tokens $u^{A}$ separate *m* construct labels and $u^{B}$ separates the concatenated construct labels from a sequence of *n* words (*w*) that constitute the item stem. The result is a string, consisting of one or multiple short descriptive labels of psychological constructs separated by delimiter tokens, followed by a statement that is indicative of those constructs (e.g., such a string might look like: “#Anxiety#Neuroticism@I worry about things”). Fine-tuning a pre-trained causal transformer model with data in this format permits later querying ${\upphi }\left( {\uppsi } \right)$ in conditional generation to return a sequence ${\upiota }$ that is heuristically related to the construct labels.

Fine-tuning the transformer to this pattern results in changes to its model weights. These shifted weights tend to represent transformations that best capture the context of the tokens before the delimiter token. How well it can do this is measured by forcing the transformer to attempt to generate the expected set of training items from the associated construct labels. The general concept of the uncertainty with regard to these attempts is termed *perplexity*, and in transformers is measured by the *cross-entropy loss*. The classification error is calculated for each token for its deviation from the predicted token and combined for the overall expected sequence. The loss is then back-propagated and the learning algorithm makes small changes to the model weights. This results in slight changes to the family of transformations it represents that grow over time into larger changes, biasing the family increasingly toward those that best encode the transformation equivalent to a very approximate form of concept elaboration. However, in practice, it works well enough to provide a practical tool for AIG.

## Workflow and Illustration

We demonstrate implicit parameterization by illustrating how training data is encoded and GPT-2 fine-tuned to the downstream task of construct-specific AIG. In doing so, we hope to guide researchers and practitioners in a tutorial-like fashion and to motivate them to explore the promising interdisciplinary domain of NLP applied to a psychometric context. Note that this procedure is expected to work similarly for any causal transformer model or more generally any autoregressive model. We recommend the use of the *transformers* Python package (Wolf et al., 2020) for fine-tuning or text generation using a wide variety of transformer models. Pretrained GPT-2 models in various sizes can be obtained via the package. At the Open Science Framework (OSF) at https://osf.io/3bh7d/, we provide an online repository with an example training data set, as well as Python code accompanying this section. Readers who wish to replicate our method will find references to source lines of code (SLOC) for fine-tuning the model (example_finetuning.py) and item generation (example_generation.py) in the remainder of this section.

**Fig. 2:**
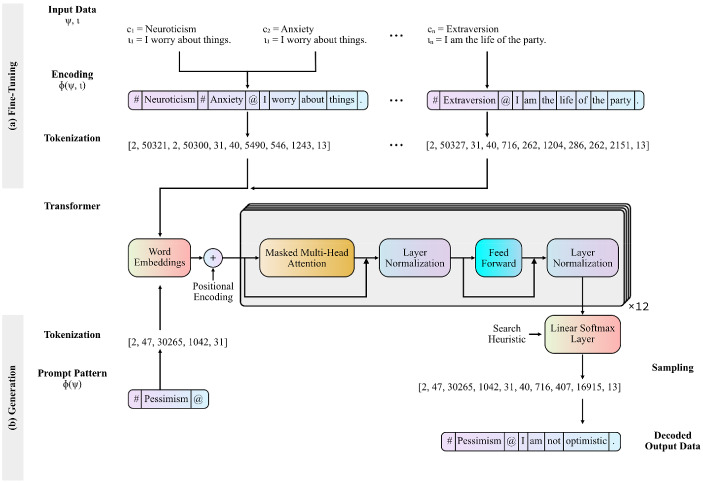
Illustration of the Workflow of the Proposed Method for Construct-Specific Automatic Item Generation. *Note*. Workflow for (a) fine-tuning a causal transformer model using the proposed segmented training pattern, and (b) applying the partial pattern to prompt a causal transformer for the generation of construct-specific item stems. The depicted transformer shows the 12-layer decoder architecture of the Generative Pretrained Transformer adopted from Radford et al. (2018), although the workflow in principle is agnostic to what causal transformer architecture is chosen.

If one wishes to fine-tune GPT-2 for the generation of construct-specific personality items, a possible large dataset of validated items must be acquired (see SLOC #27). This dataset must then be encoded according to the segmented training pattern previously described (see Eq. 6; SLOC #33). Figure 2 shows how the encoding scheme for the previously referenced exemplary items “*I am the life of the party*,” intended to assess extraversion, and “I worry about things,” intended to assess neuroticism and anxiety. As delimiter tokens we chose single ASCII characters that are infrequently used in writing.

Before commencing fine-tuning, a tokenizer is used to disassemble the encoded training data for smaller units corresponding to tokens in the models’ vocabulary (see SLOC #42). This results in a vector of integers, where each integer represents a token in the vocabulary. It may be meaningful to add all construct labels to the vocabulary in advance, so that these are learned as a single unit during fine-tuning (see SLOC #46). Considerations with regard to additional fine-tuning modalities must be made, such as determining learning rates, choosing optimization algorithms, or termination criteria but are not exclusively pertinent to language modeling and will therefore not be further discussed in this article (see SLOC #54).

**Fig. 3:**
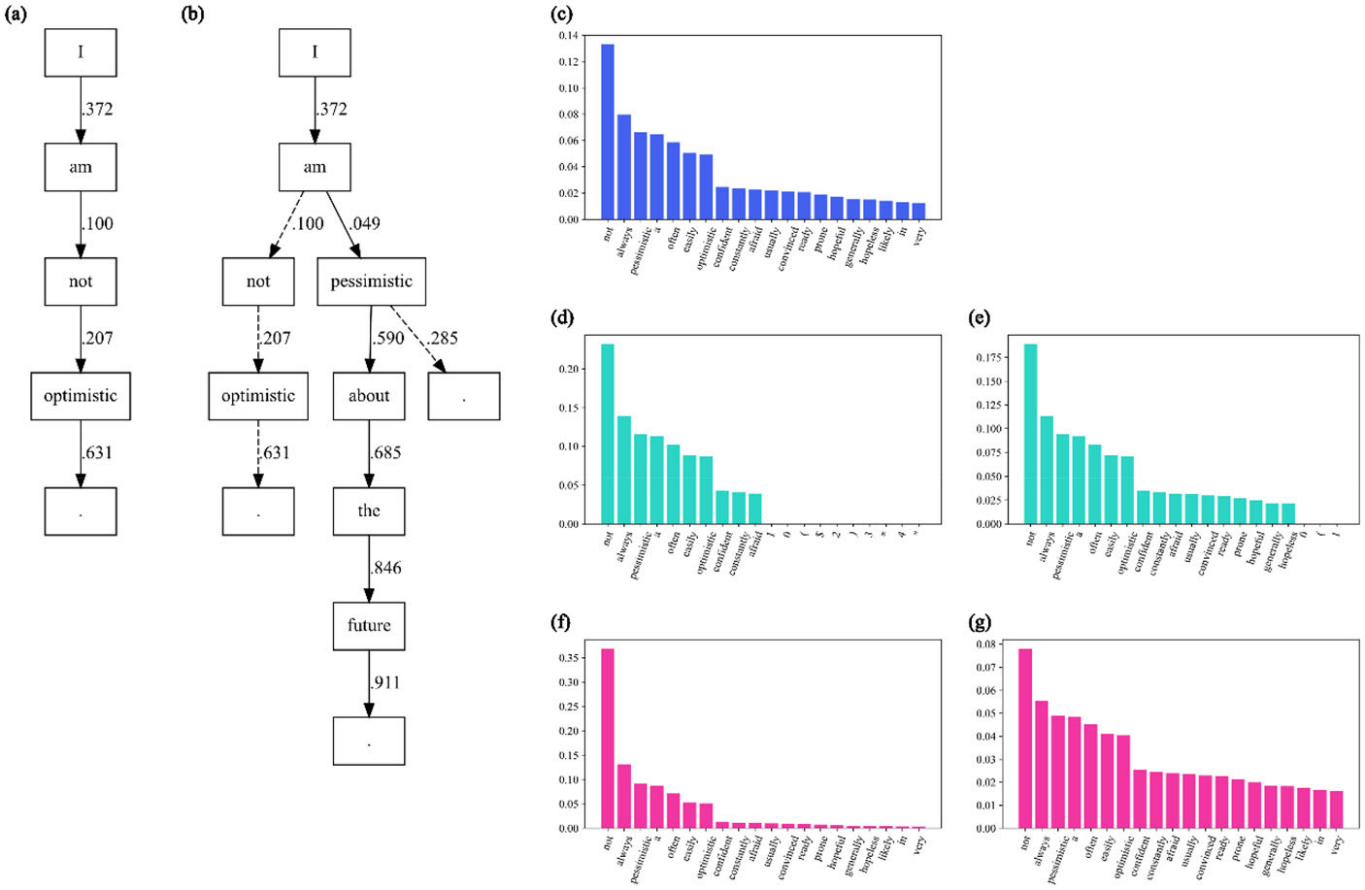
Differences in Search Heuristics for Generated Items and Tokens. *Note*. Item generation after fine-tuning when prompted for the construct label *Pessimism*, using various search heuristics. (a) greedy search; (b) beam search with $\hbox {n} = 3$ search beams, dashed lines indicate lower total sequence probabilities; (c) to (g) show next-token probabilities for the premise “*#Pessimism@I am*” on the y-axis; (c) multinomial sampling with no transformation; (d) multinomial sampling with $\textit{top-k} = 10$; (e) multinomial sampling with nucleus sampling at $\textit{top-p} = .7$; (f) multinomial sampling with temperature $= 0.5$; and (g) multinomial sampling with temperature $= 1.5$.

Once fine-tuning is performed, the partial pattern (${\upphi }\left[ {\uppsi } \right]$, see Figure 2, SLOC #13) can be used as a prompt in conditional generation. Generation will consequently yield item stems that are heuristically in the semantic vicinity of the requested construct labels, even if a requested construct label was not in the fine-tuning dataset. When using language models for text generation, multiple search heuristics can be applied that directly influence next word inference. Although a multitude of such techniques are conceivable, we will in the following discuss three frequently applied methods, namely *greedy search*, *beam search*, and *multinomial sampling*. The arguably most straightforward approach to text generation is to use a greedy search strategy (SLOC #17), in which inference is based on nothing but the highest probability token for each prediction step. For construct-specific AIG, this is the conditional probability of a word at prediction step k given a history of words that contains the linguistic manifestation of a given latent variable. Text generated using greedy search may suffer from repeating sub-sequences (Suzuki & Nagata, 2017) and may produce sentences that either lack ingenuity or exhibit an overall low joint probability. In contrast, beam search may reduce the risk of generating improbable sequences by comparing the joint probability of *n* alternative sequences (i.e., beams; SLOC #32) and selecting the overall most probable sentence (Vijayakumar et al., 2018). Figure 3 illustrates the differences in the case of construct-specific AIG for these two search heuristics.

Whereas greedy and beam search result in deterministic output and arguably fairly prototypical items, *multinomial sampling* (SLOC #49) comprises a variety of methods that accomplish text generation by sampling from the probability distribution of words, which oftentimes is transformed beforehand. In practice, this not only results in a larger pool of potential items but also mirrors human language more accurately, as argued by Holtzman et al. (2019). Multinomial sampling should be used if the goal is to generate a larger set of items.

Three common schemes are frequently used to transform the probability mass of the distribution when applying multinomial sampling. In *top-k* sampling, the probability mass for next word prediction is redistributed from the entire vocabulary to the *k* words with the highest probability (Fan et al., 2018). This effectively eliminates the risk of sampling words at the tail of the distribution while arguably permitting variations that are somewhat plausible. Nucleus sampling, also known as top-p sampling, may be used to improve the performance of top-k by allowing the cut-off to adjust dynamically to the distribution. Nucleus sampling also truncates the probability distribution, but instead of redistributing probabilities to the top k words, it prunes based on the cumulative probabilities of words before reaching a threshold (Holtzman et al., 2019). For instance, the example “e)” in Figure 3 shows a truncated probability distribution of 17 possible next-token predictions for the given prefix “*#Pessimism@I am*.” The cumulative probability of these tokens amounts to $\le 70{\%}$, thereby prohibiting that improbable will be sampled. The top-k and top-p sampling schemes, however, maintain the shape of the distribution which either may be heavily skewed and thereby too predictable, or too uniform to produce a coherent sentence or item. This can be rectified, independently from top-k or top-p sampling, by a modification to the softmax transformation (see Eq. 4) which magnifies or suppresses the modalities of the distribution by manipulating the $\uptau$ coefficient. This parameter is referred to as *temperature* (e.g., Wang et al., 2020) and is a useful utility for controlling the “creativity” of the generated output (see Figure 3). Higher values for $\uptau$ will yield a more uniform probability distribution of next-word predictions and thus favor variety.

## Empirical Study

To test the proposed method, we compared human- and machine-authored items within a questionnaire in an online survey, similar to von Davier (2018). However, the generation of construct-specific items requires additional considerations with regard to structural validity. Data, code, and generated items accompanying this study are available from https://osf.io/3bh7d/. Note that this repository also contains Python code to replicate the methods proposed in this paper. In addition, we provide a web application demonstrating construct-specific automatic item generation on https://cs-aig-server-2uogsylmbq-ey.a.run.app/1.

### Model Fine-Tuning and Item Generation

We obtained a pretrained 355 million parameter GPT-2 model with the goal of fine-tuning it to construct-specific AIG^2^. Out of the 4452 item stems and 246 construct labels in the International Personality Item Pool^3^ (Goldberg, 1999; Goldberg et al., 2006), we selected 1715 unique item stems grouped by associated construct labels with a mean of 2.40 ($\textit{SD} = 1.84$) labels for each stem. This dataset served as training data to subsequently fine-tune the 335M to the AIG task and was fed as delimited concatenated strings of construct labels and item stems as previously described in Eq. 6. Training was performed on a Nvidia GeForce RTX 2070 Super using the CUDA 9.1.85 and cuDNN 7.6.3 toolkits with TensorFlow 1.14.0 (Abadi et al., 2016) and Python 3.6.9 by an adaptation of GPT-2-Simple (Woolf, 2020) on Linux Ubuntu 18.04.4. Fine-tuning was terminated after 400 training steps with a learning rate of 5e-04 at final cross-entropy loss of 0.83. A full list of example items generated during the fine-tuning process can be found in the OSF repository.

We then prompted the model to generate item stems for two sets of construct labels in conditional generation. The first set consisted of five *trained* construct labels (*openness to experience*, *conscientiousness*, *extraversion*, *agreeableness*, and *neuroticism*) which were introduced to the model in the training dataset during fine-tuning. The second set in turn consisted of five *untrained* construct labels (i.e., *benevolence*, *egalitarianism*, *egoism*, *joviality*, and *pessimism*) that were not introduced during fine-tuning. In total, we generated 1,360 item stems associated with one of these construct labels. All items were generated using multinomial sampling with varying temperatures (0.7, 0.9; and 1.1) to increase the variability of the item pool. We refrained from using *top-k* or *top-p* sampling to sample from the full probability distribution of tokens.

### Overfit

Overfitting is a major obstacle and common phenomenon in training deep neural networks (Srivastava et al., 2014). Instead of learning abstract features, an overfitted model will tend to reproduce the original training data. We assessed an index of string similarity between the data used for model fine-tuning and the model‘s generated output as a proxy measure for model overfit. Coefficients were calculated by inverting and normalizing the Levenshtein distance (Levenshtein, 1966) between two item stems, which theoretically may range from 0 to 1, whereas the latter indicates an exact match between item stems. In essence, this metric reflects the number of single character insertions, deletions, or substitutions one must make for two strings to become identical. We regarded item stems with a similarity index $\ge .90$ as being largely identical to the training data and thus symptomatic of overfit. As most statistical thresholds are picked rather arbitrarily, we carefully chose a cut-off value based on qualitative judgment. For example, the similarity coefficient between the generated item, “I *like* to be the center of attention,” and the IPIP item, “I *love* to be the center of attention,” amounts to .95 and thus the item was discarded, whereas the similarity between “I am easily *angered*” and “I am easily *annoyed*” was below the threshold at .85. A full list of similarity indices for each generated item stem can be found in the OSF repository, including a reference to the most similar item in the training data. The mean similarity between the generated items and the most similar items in the training data was .68 ($\textit{SD} = .16$), with 164 items (12.0%) exceeding the similarity threshold of .90 and, thus, were omitted from the dataset.

### Content Validity

We further omitted duplicate items and items that were labeled with more than one construct down to a selection of 283 items. Items were subsequently rated for content validity by two independent expert judges who were carefully instructed to only rate items as valid if they (a) considered the item stem to be syntactically and linguistically correct and (b) regarded the item stem to be either clearly symptomatic or clearly asymptomatic (in case of reversed items) of the latent variable described by the construct label. The items were rated with an agreement of .72 (95% CI [.64, .80]) as indicated by Cohen’s kappa. A total of 151 (53.4%) items were endorsed by both raters for content validity. While Table S1 in the online supplemental section provides some examples of content valid and rejected items, a data file with the full list of accepted and rejected generated item stems can be found in the OSF repository.

### Questionnaire

To properly assess the psychometric properties of the generated items, we derived a Likert-style questionnaire consisting of both human- and machine-authored items. From the remaining set of 151 machine-authored items unanimously endorsed for content validity, we randomly selected 5 items for each construct label. This resulted in 25 CLIS-tuples for the five trained construct labels and 25 CLIS-tuples for the five untrained construct labels. We decided to include only a random selection of 50 items into the questionnaire to prevent fatigue in respondents and to safeguard data quality. As for the set of human-authored items, we used the 25 items from the BFI dataset in the R psych-package (version 2.0.9; Revelle, 2020, based on Goldberg, 1999; not to be confused with the Big Five Inventory by John et al., 2012). The BFI is composed of established items taken from the IPIP and reflects the Big Five factors (i.e., *openness to experience*, *conscientiousness*, *extraversion*, *agreeableness*, and *neuroticism*).

### Participants and Procedure

The final questionnaire consisted of 75 human- and machine-authored items using a 5-point Likert scale and was converted into an online survey. We recruited 273 participants through Amazon Mechanical Turk in exchange for $0.50 upon completion. Items were presented in a randomized order. We used two measures to identify and exclude potential careless responders. First, we included 3 bogus items in accordance with the recommendations by Meade and Craig (2012), which instructed participants to pick a certain response option on the presented scale. Second, we excluded participants with unreasonable response speed based on a relative-speed index $$\ge 2.0$$ (Leiner, 2019). This resulted in a final sample of 220 respondents.

**Table 1:** Comparison of Confirmatory Factor Analyses of Human- and Machine-authored Scales for Trained Construct Labels

	Human-authored						Machine-authored						
Scale	CFI	RMSEA	$\uplambda _{\mathrm{mean}}$	$\uplambda _{\mathrm{range}}$	$\upomega$	$\upomega _{\mathrm{CI}}$	CFI	RMSEA	$\uplambda _{\mathrm{mean}}$	$\uplambda _{\mathrm{range}}$	$\upomega$	$\upomega _{\mathrm{CI}}$	*p*
Openness to experience	.95	.14	.62	[.82, .72]	.72	[.65, .78]	.95	.10	.54	[.44, .75]	.66	[.66, .58]	.097
Conscientiousness	.93	.23	.72	[.74, .81]	.81	[.76, .85]	1.00	.00	.44	[.15, .69]	.46	[.46, .36]	$< .001$
Extraversion	.98	.15	.77	[.89, .86]	.86	[.82, .89]	1.00	.05	.67	[.34, .90]	.75	[.75, .68]	$< .001$
Agreeableness	.96	.17	.73	[.86, .80]	.80	[.75, .85]	.80	.27	.58	[.35, .87]	.63	[.63, .49]	$< .001$
Neuroticism	.99	.13	.80	[.91, .87]	.87	[.84, .90]	.98	.17	.56	[.02, .92]	.70	[.70, .61]	$< .001$

*Note*. $N = 220$ respondents. $\uplambda _{\mathrm{mean}} =$ Mean of standardized factor loadings; $\uplambda _{\mathrm{range}} =$ Range of standardized factor loadings; $\upomega =$ Omega coefficient of internal consistency; $\upomega _{\mathrm{CI}} =$ percentile bootstrapped 95% confidence interval for omega coefficient. $p =$ bootstrapped probability of models’ differences in omega coefficients ($K = 5,000$ bootstrapped resamples; data from $k =$ 446 iterations were omitted due to failed model convergence).

### Results

We first tested the equivalence between human- and machine-authored items for trained construct labels at the scale level. Models were computed using confirmatory factor analysis (CFA) with polychoric correlations and robust weighted least square mean and variance adjusted (WLSMV) estimators, which have been shown to produce accurate estimates for ordered categorical items with even small samples (Flora & Curran, 2004). The fit statistics are reported in Table 1. CFA model fit was overall similar for machine-authored and human-authored scales, with better fit for machine-authored conscientiousness and extraversion items and better fit for human-authored agreeableness and neuroticism items. Especially the fit for the machine-authored agreeableness scale was strikingly poor ($\hbox {CFI} = .80$, $\hbox {RMSEA} = .27$). Here we found the low fit to be due to correlated residuals between the item pairs “I care a lot about others” and “I am not a nice person” on one hand, and “I am easily angered” and “I am not easily offended” on the other. These correlated residuals can be explained by the comparatively high semantic similarity of the respective items.

We used McDonalds’s omega coefficient of internal consistency to assess reliability, which ranged between .72 (*openness to experience*, 95% CI [.65, .78]) and .87 (*neuroticism*, 95% CI [.84, .90]) for human-authored, and .46 (*conscientiousness*, 95% CI [.36, .57]) and .75 (*extraversion*, 95% CI [.68, .81]) for machine-authored items. We bootstrapped omega coefficients and corresponding confidence intervals in 5,000 iterations for each scale to compare human- and machine-authored items and found significantly smaller reliabilities for machine-authored items for all Big Five dimensions with the exception of openness to experience ($\upomega _{human} = .72$, $\upomega _{machine} = .66$, $p = .097$).

For a better understanding of the validity of specific machine-authored items, we next compared factor loadings of each individual machine-authored item when added to a model with five human-authored items of their respective scale. As depicted in Table 2, a total of 8 machine-authored items (32%) exhibited factor loadings greater or equal to those of their human-authored counterparts. Moreover, 16 items (64%) exceeded the commonly referenced cut-off value of .40 (e.g., Hinkin, 1995). In summary, we found evidence that a substantial part of the machine-authored items was as valid as human-authored items, but that other machine-authored items were not suitable at all.

**Table 2:** Descriptive Statistics and Factor Loadings of Machine-authored Items for Trained Construct Labels

Item	*M*	*SD*	Frequencies					Skewness	Kurtosis	$\uplambda$	$\in \quad \uplambda _{\mathrm{human}}$
			1	2	3	4	5				
I can enjoy a wide variety of musical styles. (OPE$+$)	4.10	1.05	7	13	30	71	99	$-1$.16	0.76	.62	1
I like to be surprised. (OPE$+$)	3.13	1.32	32	39	61	45	43	$-0$.10	$-1$.08	.36	0
I love to contemplate the universe and its beauty. (OPE$+$)	3.94	1.12	9	15	46	60	90	$-0$.87	$-0$.04	.65	1
I like to be with people who are different from myself. (OPE$+$)	3.50	1.06	9	25	75	68	43	$-0$.32	$-0$.43	.35	0
I am not a fan of change. (OPE-)	3.11	1.32	29	47	61	36	47	0.00	$-1$.13	.35	0
I am not always on time for work. (CON-)	4.01	1.28	12	28	21	43	116	$-1$.02	$-0$.29	.53	0
I know that I make many mistakes. (CON-)	2.53	1.20	53	61	57	35	14	0.35	$-0$.84	.20	0
I work too hard. (CON$+$)	3.17	1.28	25	45	62	44	44	$-0$.07	$-1$.05	.55	0
I do not like to read or study. (CON-)	4.23	1.04	8	8	27	59	118	$-1$.44	1.55	.54	0
I am not concerned with details. (CON-)	4.27	0.95	4	10	23	68	115	$-1$.39	1.57	.65	0
I am able to speak confidently. (EXT$+$)	3.96	1.11	8	18	37	69	88	$-0$.92	0.06	.84	1
I avoid public places. (EXT-)	3.50	1.28	21	31	44	66	58	$-0$.50	$-0$.85	.46	0
I am able to handle myself in a crowd. (EXT$+$)	3.98	1.07	8	15	34	79	84	$-1$.02	0.44	.73	1
I do not like to talk about myself. (EXT-)	2.59	1.25	50	65	52	32	21	0.41	$-0$.84	.45	0
I am able to hold my own in a discussion. (EXT$+$)	4.16	0.97	6	11	19	90	94	$-1$.37	1.74	.60	1
I care a lot about others. (AGR$+$)	4.25	0.92	4	5	34	67	110	$-1$.23	1.30	.87	1
I am easily angered. (AGR-)	3.96	1.17	11	19	31	65	94	$-1$.00	0.07	.39	0
I don’t like to argue. (AGR$+$)	3.95	1.14	10	17	38	65	90	$-0$.94	0.05	.23	0
I am not easily offended. (AGR$+$)	3.43	1.25	16	45	38	71	50	$-0$.36	$-1$.00	.24	0
I am not a nice person. (AGR-)	4.51	0.84	3	5	17	47	148	$-1$.95	3.83	.79	1
I am generally happy and content. (NEU-)	2.15	1.19	82	70	36	18	14	0.91	$-0$.07	.72	0
I am often upset by minor things. (NEU$+$)	2.30	1.23	72	69	33	34	12	0.64	$-0$.71	.89	1
I am a person who is easily moved by the good moods and bad moods of others. (NEU$+$)	3.47	1.23	22	24	52	73	49	$-0$.55	$-0$.63	.28	0
I am generally cheerful and optimistic. (NEU-)	2.30	1.26	72	67	43	18	20	0.76	$-0$.42	.69	0
I seldom feel scared. (NEU-)	3.01	1.28	30	57	45	56	32	0.00	$-1$.15	.38	0

*Note. * Based on data from $$ N = 220$$ respondents. $$\uplambda =$$ Standardized factor loading in a CFA model with the five human-authored items and the respective machine-authored item; $$\in \uplambda _{\mathrm{human}} =$$ Factor loading of respective machine-authored item within the range of factor loadings for human-authored scales (1 $$=$$ within the range); OPE $$=$$ Openness to experience; CON $$=$$ Conscientiousness; EXT $$=$$ Extraversion; AGR $$=$$ Agreeableness; NEU $$=$$ Neuroticism; $$+$$/- indicates positive or negative keying.

Finally, we examined machine-authored items generated for untrained construct labels. As shown in Table 3, omega coefficients indicated satisfactory to good reliability for three scales (*benevolence*; *egalitarianism*; *pessimism*), particularly when considering the small number of items per scale, and fit statistics also indicated satisfactory to good model fit. In contrast, model fit statistics and reliability estimates for *egoism* and *joviality* were not satisfactory. As shown in Table 4, at the item level a total of 19 items (76%) exceeded factor loadings of .40 in confirmatory factor analyses.

Next, we sought to discern the latent structure of the untrained item set using exploratory factor analysis (EFA) with polychoric correlations and oblique rotation. We expected that this structure would reflect a five-factor solution, corresponding to the five untrained construct labels that we had requested from the fine-tuned GPT-2 model. In line with this expectation, parallel analysis suggested a 5-factor solution. The loadings matrix of the subsequent EFA showed generally distinct loadings for conceptual items for *benevolence*, *egalitarianism* and *pessimism* (see results provided in Table S2 in the online supplemental material). The fifth factor appeared to be rather specific and absorbed items that poorly fitted to the respective conceptual scales, as indicated by relatively low proportional variance and heterogenous loading patterns.

**Table 3:** Goodness of Fit Statistics, Factor Loadings and Reliability Estimates of Confirmatory Factor Analyses of Machine-authored Scales for Untrained Construct Labels

Scale	CFI	RMSEA	$\uplambda _{\mathrm{mean}}$	$\uplambda _{\mathrm{range}}$	$\upomega$	$\upomega _{\mathrm{CI}}$
Benevolence	1.00	.05	.69	[.49, .94]	.74	[.67, .79]
Egalitarianism	.99	.09	.76	[.67, .87]	.78	[.69, .85]
Egoism	.90	.12	.44	[.08, .85]	.58	[.47, .67]
Joviality	.83	.16	.44	[.17, .92]	.54	[.42, .62]
Pessimism	.99	.11	.70	[.45, .93]	.82	[.77, .86]

*Note*. $N = 220$ respondents. $\uplambda _{\mathrm{mean}} =$ Mean of standardized factor loadings; $\uplambda _{\mathrm{range}} =$ Range of standardized factor loadings; $\upomega =$ Omega total coefficient of internal consistency; $\upomega _{\mathrm{CI}} =$ bootstrapped 95% confidence interval for omega coefficient, based on $K = 5,000$ bootstrap iterations.

**Table 4:** Descriptive Statistics and Factor Loadings of Machine-authored Items for Untrained Construct Labels

Item	*M*	*SD*	Frequencies					Skewness	Kurtosis	$\uplambda$
			1	2	3	4	5			
I care about others’ well-being. (BEN$+$)	4.41	0.76	2	1	21	77	119	$-1$.40	2.61	.78
I forgive others. (BEN$+$)	3.85	1.09	9	19	39	82	71	$-0$.85	0.05	.55
I am not a person who would do anything nice for anyone. (BEN-)	4.57	0.79	2	6	12	44	156	$-2$.15	4.71	.66
I have little sympathy for poor people. (BEN-)	4.17	1.23	14	17	16	44	129	$-1$.38	0.70	.49
I am not interested in others feelings. (BEN-)	4.30	0.98	4	11	25	55	125	$-1$.41	1.36	.94
I believe that the rights of others should be treated equally. (EGA$+$)	4.72	0.59	1	2	4	43	170	$-2$.78	10.18	.87
I believe that all races are created equal. (EGA$+$)	4.60	0.89	6	4	13	27	170	$-2$.52	6.09	.71
I believe that it is wrong to exploit others for your own gain. (EGA$+$)	4.52	0.92	7	5	9	44	155	$-2$.35	5.37	.67
I believe in the equality of all peoples. (EGA$+$)	4.65	0.72	2	3	11	38	166	$-2$.49	6.95	.81
I believe that the rights of others should be respected without question. (EGA$+$)	4.35	0.84	2	6	22	72	118	$-1$.38	1.88	.77
I believe that I have the right to my own way of life. (EGO$+$)	4.45	0.72	2	1	15	79	123	$-1$.57	3.69	.08
I often exaggerate my achievements. (EGO$+$)	1.94	1.11	97	74	25	13	11	1.24	0.84	.26
I believe that I am the best. (EGO$+$)	2.57	1.35	67	44	50	35	24	0.34	$-1$.11	.85
I believe that I have more power than others. (EGO$+$)	2.20	1.17	78	63	46	22	11	0.71	$-0$.41	.60
I am not overly proud of my achievements. (EGO-)	3.28	1.32	26	41	49	54	50	$-0$.23	$-1$.11	.39
I am very jovial. (JOV$+$)	3.37	1.18	15	37	65	57	46	$-0$.24	$-0$.83	.92
I do things that are not fun. (JOV-)	3.34	1.23	16	41	69	41	53	$-0$.12	$-0$.99	.17
I sometimes laugh out loud. (JOV$+$)	4.33	0.93	4	11	13	73	119	$-1$.61	2.39	.18
I am never sad. (JOV$+$)	1.92	1.14	106	61	26	18	9	1.15	0.40	.39
I am easily entertained. (JOV$+$)	3.62	1.06	12	17	58	88	45	$-0$.68	0.05	.55
I am not likely to succeed in my goals. (PES$+$)	1.90	1.13	110	54	33	14	9	1.15	0.46	.71
I can see that things are never going to be the way I want them to be. (PES$+$)	2.72	1.33	51	50	57	33	29	0.26	$-1$.05	.52
I am not optimistic. (PES$+$)	2.09	1.28	103	49	26	29	13	0.88	$-0$.51	.93
I am always on the lookout for a better way. (PES-)	1.99	0.97	79	83	44	9	5	0.90	0.55	.45
I look at the bright side. (PES-)	2.23	1.25	79	69	32	23	17	0.83	$-0$.39	0.90

*Note.* Based on data from $$ N = 220$$ respondents. $$\uplambda =$$ Standardized factor loadings in a CFA model including the five machine-authored items of the respective dimension; BEN $$=$$ Benevolence; EGA $$=$$ Egalitarianism; EGO $$=$$ Egoism; JOV $$=$$ Joviality; PES $$=$$ Pessimism; $$+$$/- indicates positive or negative keying.

## Discussion

This paper offers a comprehensive examination of how deep learning language modeling can be used to automatically generate valid personality items that measure specific constructs. To achieve this, we utilized a popular pretrained transformer model, GPT-2, by fine-tuning it using the International Personality Item Pool (Goldberg et al., 2006). In doing so, we expand on work by von Davier (2018) in which Long Short-Term Memory Models were trained to create syntactically correct items.

Our primary contribution emphasizes construct-specific automated item generation, showing that it is possible to align item stems to specific constructs and to classify unconditionally generated item stems with correct construct labels. To achieve this, we taught GPT-2 a pattern by concatenating strings of personality statements with labels corresponding to constructs for which the items were conceptualized. By learning this pattern, we anticipated that the model would respond by generating valid item stems when prompted by a given construct label. We considered this task to be the inverse problem of text summarization since it requires a model to elaborate on a concept. As we outlined in the introductory section of this paper, this can only be achieved by language models which are able to learn the relationship between words beyond close proximity. Transformer models excel at long-distance dependencies and it is conceivable that GPT-2 is the first model that is capable of the construct-specific generation of personality items. The ability to adapt to patterns such as the segmented training pattern used in this paper is an important prerequisite for AIG because it permits an agent to exert control over the generated output after fine-tuning is completed. The successful adaptation of GPT-2 to the segmented training pattern therefore not only fulfills the basic requirements for meaningful AIG applications, but also implies that additional perhaps more complex patterns could be learned.

In addition to this conceptual contribution, we conducted an empirical study to examine how automatically generated items fared when assembled into a personality questionnaire. We studied two groups of items to test the structural validity of machine-authored items. One set consisted of items generated for construct labels which GPT-2 had learned during fine-tuning, while the other set comprised items authored for construct labels that were not introduced earlier. Our results showed that neither set of items is comparable in structural validity to what should be expected from a psychometrically sound personality questionnaire. Yet approximately one third of the machine-authored items for untrained construct labels showed sizable factor loadings in the same range as those of human-authored items of the same scale. More than half of these items even met or exceeded cut-off values commonly used by scale developers. Additionally, several items of the set of items generated for untrained construct labels exhibited satisfactory scale statistics. For example, 76% showed factor loadings above .40 and in three out of five scales, internal consistency exceeded coefficients of .70. Considering that generated items were in competition with items developed through years of research, we deem these results highly encouraging.

### Limitations

Although the capabilities of modern pretrained causal transformers are quite formidable, some restrictions remain that limit their applicability to AIG. Most notably, the quality of items generated with our method is currently difficult to predict. As some items generated by our model were qualitatively and psychometrically inferior to human-authored items, any practical application would currently require expert oversight. This is also necessary to avoid that semantically very similar items are selected, a problem that we observed in our study for the agreeableness scale, and which resulted in poor model fit due to correlated residuals. Human-in-the-loop systems are quite common in machine learning (Chai & Li, 2020) and may be a tolerable transitional solution. This problem could perhaps be remedied by automatically evaluating semantic similarity in post-processing. Next, generated items tend to contravene item writing guidelines and psychometric principles. As such, we have frequently seen fine-tuned models phrase double-barreled items, use negations, or conflate multiple constructs within one item, violating unidimensionality (Nunnally & Bernstein, 1994). Perhaps this could be remedied by training a bidirectional classifier model (e.g., a BERT-network; Devlin et al., 2018) to detect such violations. Such a penalty could be integrated in the loss-function when fine-tuning a language model to AIG. Moreover, we identified inadequate item difficulty as a dominant reason for poor item and scale statistics in machine-authored items. For example, all items generated for the egalitarianism construct label were overwhelmingly endorsed by respondents. Extreme difficulty is a likely symptom of a variety of potential causes, such as statements that are socially undesirable to endorse or reject (e.g., “*I believe that all races are created equal*”). It is important to find ways to gain control over these aspects to advance this line of research and to make practical applications of AIG feasible.

While our proposed method solves concept elaboration in the case of AIG in the domain of personality, we have not offered any tangible advice on how the process of fine-tuning causal transformers can be optimized to improve our results. Here, a variety of enhancement measures are conceivable. In light of the dearth of openly accessible training data in the domain of personality testing, perhaps data augmentation techniques similar to those conventionally applied in image recognition can be applied (Perez & Wang, 2017). Moreover, researchers could attempt to optimize the fine-tuning process more directly, perhaps by modifying the objective function of the neural network or by freezing the lower layers of the transformer (Lee et al., 2019; Lu et al., 2021).

On a more fundamental level, another obstacle is that we remain oblivious to the true size of the problem space. As such, it is currently not possible to estimate the limits of GPT-2—or any other causal transformer model—with regard to our notion of concept elaboration. One simply cannot know in advance what level of precision or proportion of validity that can be achieved by current technology given better training strategies or better training data. In addition, although we advocated the use of multinomial sampling for the generation of larger item pools, techniques must be derived to estimate the size of the universe of possible meaningful items that can be obtained from a model. In essence, since there is no theoretical reason to assume that probabilistic language models per se should be inferior to human test developers, deficiencies in item generation can only be attributed to model architecture, pretrained model parameters, and fine-tuning. Since the proportion of each of these components is likely to remain unknown, it is difficult to judge how close our results come to a model-specific optimum. This is problematic since it leaves future researchers without means to determine if stagnation is due to inadequate methodology with regard to model fine-tuning or because a language models’ potential has been exhausted.

### Future Directions for the Automatic Generation of Non-cognitive Items

Future developments in deep language modeling will likely continue to benefit research and assessment technology for sequence-based AIG for personality items. As noted by a reviewer, one might wonder in what use case it is desirable to obtain large quantities of personality items. The primarily current practical utility of our proposed method is limited to a decision support system (Rosenbusch et al., 2020) for item authors, which in some cases may lessen the dependence on content specialists. When constructing a scale, authors require a large item pool from which they can select items with the best psychometric properties to cover the full breadth of a target construct. Even larger quantities of items are required in computerized adaptive testing (CAT), where test developers may use our approach with multinominal sampling, to obtain a large variety of potential items. Language models for non-cognitive AIG may be a valuable tool to expand the original item pool, improving the quality of scales. We demonstrate this use case by offering an easy-to-use internet tool at https://cs-aig-server-2uogsylmbq-ey.a.run.app/ for creating items for a given construct, which can be used by scale authors without knowledge of computer science or AIG.

Furthermore, it is important to note that deep language models not merely generate text, but also derive embeddings that encode a richness of abstract information about the generated item. Operations on such vectors could lead to a host of potential improvements in scale development. For example, measures of semantic similarity (Kjell et al., 2019; Rosenbusch et al., 2020) could be integrated in the loss-function of a transformer model or perhaps even explicitly prompted to enable test developers to specify a desirable distance to a target construct. This could permit psychometricians to control content coverage a priori to item development.

While our research demonstrates that *implicit* parameterization can be used for item generation at the construct level, future work should attempt to expand on such parameterization to include psychometric properties. The highly promising prospect of using CAT in conjunction with AIG has previously been discussed in the literature (Glas & van der Linden, 2003; Simms et al., 2011; Luecht, 2013). Sentence embeddings offer a potential extension of CAT to the domain of personality item generation, if difficulty estimates could be extracted from such embeddings. When this is achieved, it is conceivable that personality questionnaires could be assembled “just-in-time,” tailored to the individual test-taker, instead of maintaining large, static item banks, as usually required for CAT. This goal, distant as it currently may seem, may help guide the future research agenda in the field of non-cognitive AIG. Such an agenda should primarily focus on two aspects:

First, language models must reliably produce valid items. In contrast to template-based AIG techniques, this is more difficult to attain when using probabilistic language models. Indeed, Bejar (2013) noted that “item generation and construct representation go hand in hand” (p. 43). This is much closer to the truth when using strictly algorithmic approaches to AIG, rooted in conventional item modeling (Gierl et al., 2008). The heuristic nature of pretrained language models, however, obscures the relationship between output and construct, rendering such methods exceedingly unpredictable. In order to use just-in-time AIG in conjunction with CAT, it is imperative that the item generating method—in our case language models —reliably produce items that represent a requested construct, i.e., hold validity, without exceptions. This may be achieved by modifications to the model architecture, larger pretrained models, or better and larger quantities of training data.

Second, future AIG techniques must permit control over latent parameters such as item difficulty, measurement invariance, or even face validity. As illustrated by some items generated within the scope of our empirical study, the proportion of socially desirable items was tremendously high. Such levels of item difficulty are rarely desirable in psychometric testing. Naturally, in contrast to static item banks used for CAT which contain information about item difficulty, a just-in-time generated item used for the same purposes must be precalibrated to specific difficulty levels prior to its creation.

Besides such general improvements, we would welcome the application of language modelling to other test formats that have not been addressed by conventional AIG techniques to date. Certainly, situational judgment tests (Lievens et al., 2008), forced-choice response formats (Cao & Drasgow, 2019), and conditional reasoning tests (James, 1998) could also benefit from the potential that lies within modern approaches to language modeling.

## Supplementary Information

Below is the link to the electronic supplementary material.

Supplementary file 1 (pdf 168 KB)

## Footnotes

1 An up-to-date link is provided in the online repository.

2 Retrieved April 28, 2020, from https://storage.googleapis.com/gpt-2/models/335M/viahttps://github.com/openai/gpt-2/blob/master/download_model.py

3 Retrieved on the April 22, 2020, from https://ipip.ori.org/
