# Supplementary material for: Transformer-Based Deep Neural Language Modeling for Construct-Specific Automatic Item Generation
Source: Psychometrika. 2021 Dec 14;87(2):749–72. doi: 10.1007/s11336-021-09823-9 (PMC9166894; doi:10.1007/s11336-021-09823-9)
Supplement: Supplementary file 1 — (pdf 168 KB) [file 11336_2021_9823_MOESM1_ESM.pdf]

Supplemental Material for the manuscript entitled

“Transformer-Based Deep Neural Language Modeling for Construct-Specific Automatic Item Generation”

Further information, as well as data and code, are available at the Open Science Framework,

https://osf.io/3bh7d/

Table S1

Examples of Endorsed and Rejected Machine-Authored Items in Content Validity Rating

	Endorsed	Rejected
Openness to Experience	I like to experience new things.	I love to be in nature.
Conscientiousness	I don't bother to read the fine print of a contract.	I have an intense desire to know the truth.
Extraversion	I avoid public places. (R)	I show a lot of my body.
Agreeableness	I have an extremely negative view of others. (R)	I have an unusually warm or fuzzy feeling when I look at someone.
Neuroticism	I am often upset by minor things.	I am often happy, even though I know I am not.
Benevolence	I have little sympathy for poor people. (R)	I have a cold.
Egalitarianism	I believe that all people should have equal rights.	I believe that all should live in harmony.
Egoism	I have an exaggerated sense of my own importance.	I didn't think that way.
Joviality	I laugh often.	I have a good time talking about the weather.
Pessimism	I believe that the future is bleak.	I see things my way.

Note. Excerpt from N = 1,360 generated items, showing typical examples of personality items endorsed for content validity or rejected during the rating process. R = Negatively keyed items.

Table S2

Exploratory Factor Analysis Results of Machine-authored Items for Untrained Construct Labels

Items	Factor Loadings				
	1	2	3	4	5
I care about others' well-being. (BEN+)	.12	.07	**.82**	.14	-.06
I forgive others. (BEN+)	.34	.03	**.48**	-.02	.23
I am not a person who would do anything nice for anyone. (BEN-)	-.03	.13	**.46**	-.35	.04
I have little sympathy for poor people. (BEN-)	-.33	.25	**.35**	-.32	.04
I am not interested in others feelings. (BEN-)	-.05	.02	**.88**	-.03	.03
I believe that the rights of others should be treated equally. (EGA+)	-.03	**.83**	.02	-.07	-.06
I believe that all races are created equal. (EGA+)	.03	**.72**	-.07	.04	.06
I believe that it is wrong to exploit others for your own gain. (EGA+)	-.14	**.53**	.26	-.09	-.17
I believe in the equality of all peoples. (EGA+)	.01	**.83**	.12	.17	-.05
I believe that the rights of others should be respected without question. (EGA+)	.00	**.79**	-.04	-.04	.16
I believe that I have the right to my own way of life. (EGO+)	.21	**.47**	-.19	.00	-.43
I often exaggerate my achievements. (EGO+)	.23	-.08	.03	**.67**	.03
I believe that I am the best. (EGO+)	**.78**	-.02	-.13	.11	.17
I believe that I have more power than others. (EGO+)	**.65**	-.07	-.13	.26	-.04
I am not overly proud of my achievements. (EGO-)	**.45**	.08	.05	-.14	.16
I am very jovial. (JOV+)	**.63**	-.02	.25	-.02	-.06
I do things that are not fun. (JOV-)	.20	.09	.10	-.05	**.62**
I sometimes laugh out loud. (JOV+)	.13	.04	.16	-.20	**-.52**

Items	Factor Loadings				
	1	2	3	4	5
I am never sad. (JOV+)	**.45**	.05	-.21	-.18	.20
I am easily entertained. (JOV+)	**.63**	-.02	.15	-.03	-.19
I am not likely to succeed in my goals. (PES+)	**-.53**	-.08	-.11	.36	.07
I can see that things are never going to be the way I want them to be. (PES+)	-.19	.13	.07	**.75**	.02
I am not optimistic. (PES+)	**-.53**	.00	-.22	.41	-.05
I am always on the lookout for a better way. (PES-)	**-.43**	-.39	.00	.05	.06
I look at the bright side. (PES-)	**-.65**	-.12	-.23	.26	-.01

Note. *N* = 220. Oblique rotation with polychoric correlations were used. Highest factor loadings on each component are in bold. CS = component solution. BEN = Benevolence; EGA = Egalitarianism; EGO = Egoism; JOV = Joviality; PES = Pessimism; +/- indicates positive or negative keying.
